# The reporting quality of natural language processing studies: systematic review of studies of radiology reports

**DOI:** 10.1186/s12880-021-00671-8

**Published:** 2021-10-02

**Authors:** Emma M. Davidson, Michael T. C. Poon, Arlene Casey, Andreas Grivas, Daniel Duma, Hang Dong, Víctor Suárez-Paniagua, Claire Grover, Richard Tobin, Heather Whalley, Honghan Wu, Beatrice Alex, William Whiteley

**Affiliations:** 1 Centre for Clinical Brain Sciences, University of Edinburgh, Chancellor’s Building, Little France, Edinburgh, EH16 4TJ Scotland, UK; 2 Centre for Medical Informatics, Usher Institute, University of Edinburgh, Edinburgh, Scotland, UK; 3 Brain Tumour Centre of Excellence, Cancer Research UK Edinburgh Centre, University of Edinburgh, Edinburgh, Scotland, UK; 4 School of Literatures, Languages and Cultures (LLC), University of Edinburgh, Edinburgh, Scotland, UK; 5 Institute for Language, Cognition and Computation, School of Informatics, University of Edinburgh, Edinburgh, Scotland, UK; 6 Health Data Research UK, London, UK; 7 Division of Psychiatry, University of Edinburgh, Edinburgh, UK; 8 Institute of Health Informatics, University College London, London, UK; 9 Edinburgh Futures Institute, University of Edinburgh, Edinburgh, Scotland, UK; 10 Nuffield Department of Population Health, University of Oxford, Oxford, UK

**Keywords:** Natural language processing, Radiology reports, Systematic review

## Abstract

**Background:**

Automated language analysis of radiology reports using natural language processing (NLP) can provide valuable information on patients’ health and disease. With its rapid development, NLP studies should have transparent methodology to allow comparison of approaches and reproducibility. This systematic review aims to summarise the characteristics and reporting quality of studies applying NLP to radiology reports.

**Methods:**

We searched Google Scholar for studies published in English that applied NLP to radiology reports of any imaging modality between January 2015 and October 2019. At least two reviewers independently performed screening and completed data extraction. We specified 15 criteria relating to data source, datasets, ground truth, outcomes, and reproducibility for quality assessment. The primary NLP performance measures were precision, recall and F1 score.

**Results:**

Of the 4,836 records retrieved, we included 164 studies that used NLP on radiology reports. The commonest clinical applications of NLP were disease information or classification (28%) and diagnostic surveillance (27.4%). Most studies used English radiology reports (86%). Reports from mixed imaging modalities were used in 28% of the studies. Oncology (24%) was the most frequent disease area. Most studies had dataset size > 200 (85.4%) but the proportion of studies that described their annotated, training, validation, and test set were 67.1%, 63.4%, 45.7%, and 67.7% respectively. About half of the studies reported precision (48.8%) and recall (53.7%). Few studies reported external validation performed (10.8%), data availability (8.5%) and code availability (9.1%). There was no pattern of performance associated with the overall reporting quality.

**Conclusions:**

There is a range of potential clinical applications for NLP of radiology reports in health services and research. However, we found suboptimal reporting quality that precludes comparison, reproducibility, and replication. Our results support the need for development of reporting standards specific to clinical NLP studies.

**Supplementary Information:**

The online version contains supplementary material available at 10.1186/s12880-021-00671-8.

## Background

Medical imaging reports, written by radiologists, contain rich data about patients’ health and disease which are not routinely captured in structured healthcare administrative datasets. Ready access to these data would be of great benefit for research and health-care quality improvement, particularly to examine the health of large populations. However, this resource is currently underutilised because manual extraction of data from free-text imaging reports is time-consuming. Natural language processing (NLP) is an automated technique used to analyse language (often in free-text) and convert it to a structured format that is easier to use; thus, NLP provides the means to retrieve granular information from imaging reports [1], by-passing the need for manual extraction, and simplifies research with these data.

Systematic review of the clinical NLP literature is important to identify promising developments, potential harms, and to help avoid duplication of effort; however, research synthesis in this area is complicated by a lack of consistency in study methods and reporting [2]. There are no clear reporting guidelines for clinical NLP studies, perhaps because NLP is used in so many different study designs. Methods and reporting guidance for clinical trials using machine learning (ML) [3–5] have recently been published, and extended guidelines are also being developed for the reporting of predictive ML models [6, 7]. Structured reporting protocols have also been suggested for NLP in clinical outcomes research [8] and also codes of practice for the use of Artificial Intelligence (AI) in radiology [9]. However, publications which have evaluated the reporting standards of ML studies [6] and its sub-field, deep learning (DL) [10], in clinical settings have shown low reporting standards which make this research difficult to interpret, replicate, or synthesise. Whether clinical NLP in general has better reporting is unclear from existing reviews [11].

In this systematic review, we examine the quality of reporting of studies that apply clinical NLP to imaging reports. We chose imaging reports because they are relatively accessible and of small size, with a restricted vocabulary [12], which makes them suitable for NLP. We aimed to establish the current state of reporting of studies that apply NLP to imaging reports and to identify NLP-specific criteria to assist future reporting. An accompanying informatics paper has been written which provides a more detailed overview of the NLP methods used and their clinical applications [13].

## Methods

We published our review protocol (10.17504/protocols.io.bmwhk7b6) [14] and this report follows the Preferred Reporting Items for Systematic Reviews and Meta-Analysis (PRISMA) [15] guideline.

### Search strategy

We designed an automated search of Google Scholar with 'Publish or Perish' software [16] to identify articles published between January 2015 and October 2019, building on an existing review by Pons et al. [11] which included literature published up to October 2014 (details of the automated search can be found in Additional file 1). Our search was executed on 27th November 2019 and our search terms were: ("radiology" OR "radiologist") AND ("natural language" OR "text mining" OR "information extraction" OR "document classification" OR "word2vec") NOT patent. We also used a snowballing method to conduct a citation search using a list of publications that cite the Pons et al. review [11] and the articles cited in Pons’ review [11]. The results of these two search approaches were combined [13].

### Study selection

We first ran an automated screening of papers to remove any duplicates and irrelevant publications. The criteria used to filter out irrelevant publications were: language is not English; the word 'patent' is found in the title or URL; year of publication before 2015, as our review aimed to update a previous review by Pons et al. (2016); the words 'review' or 'overview' found in the title, or 'this review' found in the abstract; image keywords found in the title or abstract with no NLP terminology in the abstract; and finding either no radiology keywords or no NLP terminology in the title or abstract (more details can be found in Additional file 1).

Four reviewers (three NLP researchers [DD, AG, HD] and one epidemiologist [MTCP]) then screened all titles and abstracts for potentially eligible studies. All papers that two or more reviewers approved for inclusion progressed to full paper review, and papers only selected by one reviewer for inclusion were discussed by these four reviewers to achieve agreement on inclusion or exclusion. Lastly, eight reviewers (six NLP researchers [AG, HD, VS, AC, BA, HW] and two epidemiologists [ED and MP]) carried out the full paper screening according to the inclusion and exclusion criteria specified below and resolved any uncertainties by group discussion. All papers were double reviewed by an NLP researcher.

### Inclusion and exclusion criteria

We included studies that applied NLP to radiology reports of any imaging modality. Our exclusion criteria were: (1) wrong publication type e.g. case reports, reviews, conference abstracts, comments, or editorials; (2) research not using radiology reports (e.g. using lab reports or clinical notes); (3) research using radiology images only (not using NLP methods); (4) not reporting any NLP results; (5) not available in full text; (6) duplicates; (7) articles written in a language other than English; (8) published before 2015; and (9) patents. The last four criteria should have been pre-filtered out by the automatic screening but we maintained these criteria to be consistent and exclude any papers that the filtering had missed.

### Data extraction

The key data extracted were: year of publication, primary clinical application and primary technical objective, study period, language of radiology reports, anatomical region, imaging modality, disease area, size of data set, annotated set size, training set size, validation set size, test set size, external validation performed, domain expert used, number of annotators, inter-annotator agreement, NLP technique(s) used, best reported results (recall, precision and F1 score), availability of data set, and availability of code.

### Data categorisation

We categorised the primary clinical application of each study. ‘Clinical application’ was the reported health-related purpose of the study. We iteratively developed a classification to represent the literature in our review, extending an existing categorisation [11], which ultimately included the categories of diagnostic surveillance, disease information and classification, language discovery and knowledge structure, quality and compliance, cohort and epidemiology, and technical NLP (Table 1).

**Table 1:** Clinical application areas and their definitions

Application area (number of papers)	Description	Subcategory	Description of included papers	Technical task (number of papers)	Anatomical scan region [Paper number—a full list of the papers by number is included in Additional file 1]
Surveillance (45)	Using imaging reports for surveillance of disease at a population health or individual level either longitudinally or generating alerts	Disease surveillance	Monitoring occurrence of infectious disease Monitoring non-communicable disease patterns, including alerts for conditions	*Information Extraction (3)*	Thorax [25] Mixed [26] Cerebrovascular [19]
				*Classification (4)*	Thorax [27, 28] Abdomen [29] Mixed [30]
		Prioritising reports	Generating alerts for reports requiring more urgent action	*Classification (4)*	Other [31] Mixed [32] Unspecified [33, 34]
		Incidental findings	Generating alerts for incidental findings	*Information Extraction (1)*	Mixed [23]
				*Classification (3)*	Thorax [20, 21] Cerebrovascular [24]
		Patient surveillance	Pairing measurements and linking reports to Investigate/monitor conditions over time e.g. worsening prognosis/ response to treatment	*Information Extraction (21)*	Thorax [35] Abdomen [6, 8, 16–18] Mixed [1, 2, 9, 36, 37, 131] Breast [3, 10, 11, 13–15] Unspecified [7, 38, 39]
				Classification (5)	Thorax [4, 40] Breast [12] Mixed [5, 41]
		Follow-up	Detecting follow-up recommendations, creating alerts and linking to see if carried out	Information Extraction (3)	Abdomen [42] Unspecified [43] Mixed [44]
				Classification (1)	Unspecified [45]
Disease information and classification (46)	Using imaging reports to identify information that may also be aggregated according to classification systems (no specific clinical purpose specified)	N/A	Extracting information about a disease/condition/function (e.g. LVEF) (no additional processing required) Staging e.g. using BIRADS or Lung-RADS Identifying sub-types of disease Classification of fractures Predicting ICD codes ICD codes used for ground truth	Information Extraction (14)	Cerebrovascular [47,54,55] Breast [59,65] Abdomen [66] Thorax [63,67–70] Mixed [71,72] Unspecified [73]
				Classification (32)	Cerebrovascular [46, 48–53, 74–76] Abdomen [77] Breast [56–58] Extremities [78–84] Mixed [85–89] Spine [22, 90] Thorax [60–62, 64]
Language discovery and knowledge structure (27)	Investigating the structure of language in imaging reports and ways in which this may be optimised to facilitate knowledge and decision support, communication (both internally between clinicians and outward facing communication with patients/public), and assist in improving NLP applications	Knowledge support for patients/public	Improving readability of reports and communications for public/patients	*Lexicon/ontology discovery (3)*	Mixed [91, 92, 99]
		Knowledge and decision support for clinicians	Providing information for clinician use (including using ontologies and lexicons) Finding relevant reports Improving reading efficiency Supporting radiological and clinical decision making Supporting clinician education	*Clustering (1)*	Mixed [100]
				*Information Extraction (8)*	Breast [94] Mixed [101–104] Thorax [93] Cerebrovascular [105] Unspecified [106]
				*Lexicon/Ontology (1)*	Unspecified [107]
		Variability, complexity and structure of language for NLP purposes	Investigating variability and complexity of language including free-test and structured reports Improving structure of language for NLP e.g. normalising phrases to support classification Normalising and disease specific phrases	*Information Extraction (6)*	Thorax [96, 108] Mixed [97, 109, 110] Unspecified [98]
				*Lexicon/ontology discovery (4)*	Unspecified [111, 112] Spine [113] Breast [114]
				*Classification (4)*	Unspecified [95] Thorax) [115–117]
Quality and compliance (20)	Using imaging reports to assess quality and safety of radiology practice, clinical practice, and efficiency of healthcare services	Assessing imaging practices	Do imaging practices adhere to guidance including indications and protocol selection Impact of guideline changes on imaging practice Assessing imaging utilisation and yield	Information Extraction (4)	Abdomen [121] Thorax [122] Mixed [119, 123]
				Classification (11)	Mixed [118, 120, 125, 135] Cerebrovascular [124, 126, 127] Thorax [128, 129] Abdomen [130] Extremities [136]
		Audit	Classification used for quality improvement in radiology and clinical practice Identifying reports for auditing Identifying and fixing errors in reports (e.g. gender/laterality)	Information Extraction (2)	Mixed [134] Cerebrovascular [133]
				Classification (3)	Thorax [132] Breast [137] Extremities [138]
Research (16)	Using imaging reports to create patient cohorts for research purposes	Cohort	Identifying cohorts for research purposes with specific medical conditions (sometimes in specified anatomical regions), with particular radiological findings, who have had certain healthcare interactions	Classification (7)	Abdomen [140–142] Cerebrovascular [144, 152] Mixed [151] Spine [150]
				Information Extraction (3)	Unspecified [153] Cerebrovascular [145] Spine [143]
		Epidemiology	Identifying research cohorts as above but papers in which they go on to do epidemiological analyses and present their results	Information extraction (4)	Unspecified [154] Cerebrovascular [148] Abdomen [139] Thorax [147]
				Classification (2)	Mixed [146] Thorax [149]
Technical NLP (10)	Papers which do not fit to a specific category, often with a primarily technical aim	N/A	Studies encompassed a variety of purposes, such as negation detection, spelling correction, fact checking, methods for sample selection, crowd source annotation	Information Extraction (6)	Mixed [155,157,159] Thorax [160] Unspecified [161, 162]
				Classification (4)	Cerebrovascular [158] Mixed [163] Thorax [156] Unspecified [164]

### Quality assessment

There are no reporting guidelines or risk of bias tools available specifically for clinical NLP studies. To address this issue, we specified 15 criteria which we considered would need to be reported to enable assessment of risk of bias and assist replication of these studies. We took account of both existing guidelines for epidemiological research [17] and also guidance emerging from the clinical NLP community [3–9] when selecting these criteria, and sought group consensus on items that were generic measures of quality that would be readily applicable across the broad selection of methods included under clinical NLP. These criteria are described in detail in Table 2 and fall under the five headings of: data source, datasets, ground truth, outcomes, and reproducibility. Our choice of criteria may not encompass everything necessary to assess all NLP studies in radiology. For example, there may be additional outcomes metrics that need to be reported (other than precision and recall) depending on the NLP tasks and clinical applications. There also may be additional, more specific, measures that would further assist reproduction and allow comparison of the performance of particular types of NLP, such as hyperparameter selection for ML [18]. However, as we included a broad remit of research, across ML, DL and Rule Based systems, we were unable to include such granular measures specific to any particular method. However, our criteria represent core considerations identified to allow a consistent overview of the quality of studies across the heterogeneous body of research comprising clinical NLP and could be further developed for specific methods.

**Table 2:** Items used to assess the quality of reporting criteria in the current review

Quality heading	Quality criteria	Definition
Data source	(1) Sampling	Reported details of the sampling strategy for radiology reports, including whether they are from consecutive patients
	(2) Consistent imaging acquisition	Reported whether radiology reports were from images taken from one imaging machine or more and, if more, whether these machines were of comparable specification
Dataset criteria	(3) Dataset size	Reported their dataset size of > 200
	(4) Training dataset	Reported training data set size—the part of the initial dataset used to develop an NLP algorithm
	(5) Test dataset	Reported test data set size—part of the initial dataset used to evaluate an NLP algorithm
	(6) Validation dataset	Reported validation data set size—a separate dataset used to evaluate the performance of an NLP algorithm in a clinical setting (may be internal or external to the initial dataset)
Ground truth criteria	(7) Annotated dataset	Reported annotated data set size—data which has been marked-up by humans for ground truth
	(8) Domain expert for annotation	Reported use of a domain expert for annotation—annotation carried out by a radiologist or specialist clinician
	(9) Number of annotators	Reported the number of annotators
	(10) Inter-annotator agreement	Reported the agreement between annotators (if more than one annotator used)
Outcome criteria	(11) Precision	Reported precision (positive predictive value)
	(12) Recall	Reported recall (sensitivity)
Reproducibility criteria	(13) External validation	Reported whether the NLP algorithm is tested on external data from another setting (a separate healthcare system, hospital or institution)
	(14) Availability of data	Reported whether their data set is available for use (preferably with link provided in paper)
	(15) Availability of NLP code	Reported whether their NLP code is available for use (preferably with link provided in paper)

### Assessment of performance

We did not summarise results quantitatively due to the anticipated methodological heterogeneity. Our approach was a narrative synthesis of studies and visual summarisation of NLP performance stratified by quality of reporting and clinical application categories. We categorised studies into high and low reporting quality groups by the median number of qualities achieved. For this analysis, when a study reported precision and recall without F1 score, we derived the corresponding F1 score for summarisation.

## Results

### Study selection and characteristics

**Fig. 1:**
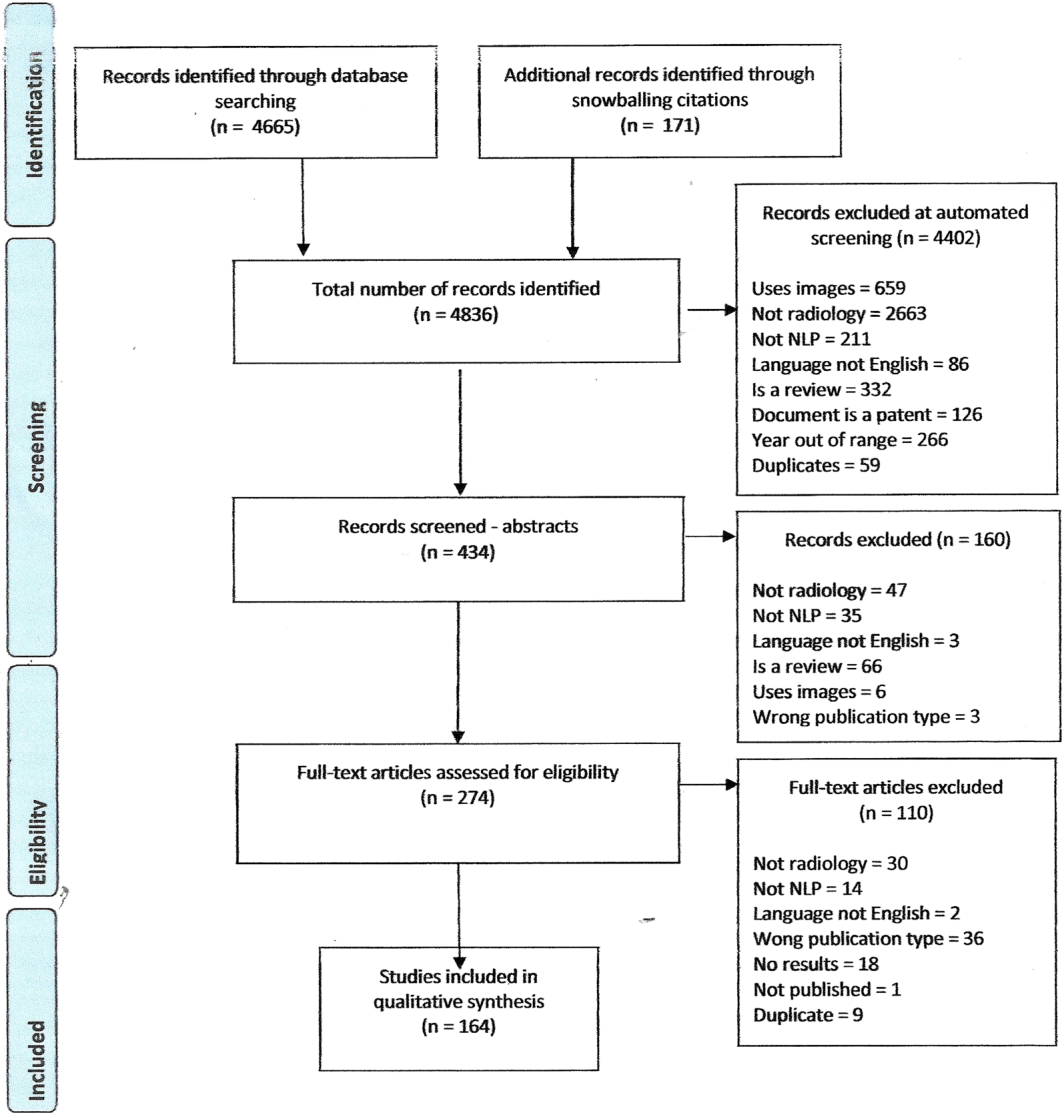
PRISMA flowchart outlining the study selection process [13]

**Fig. 2:**
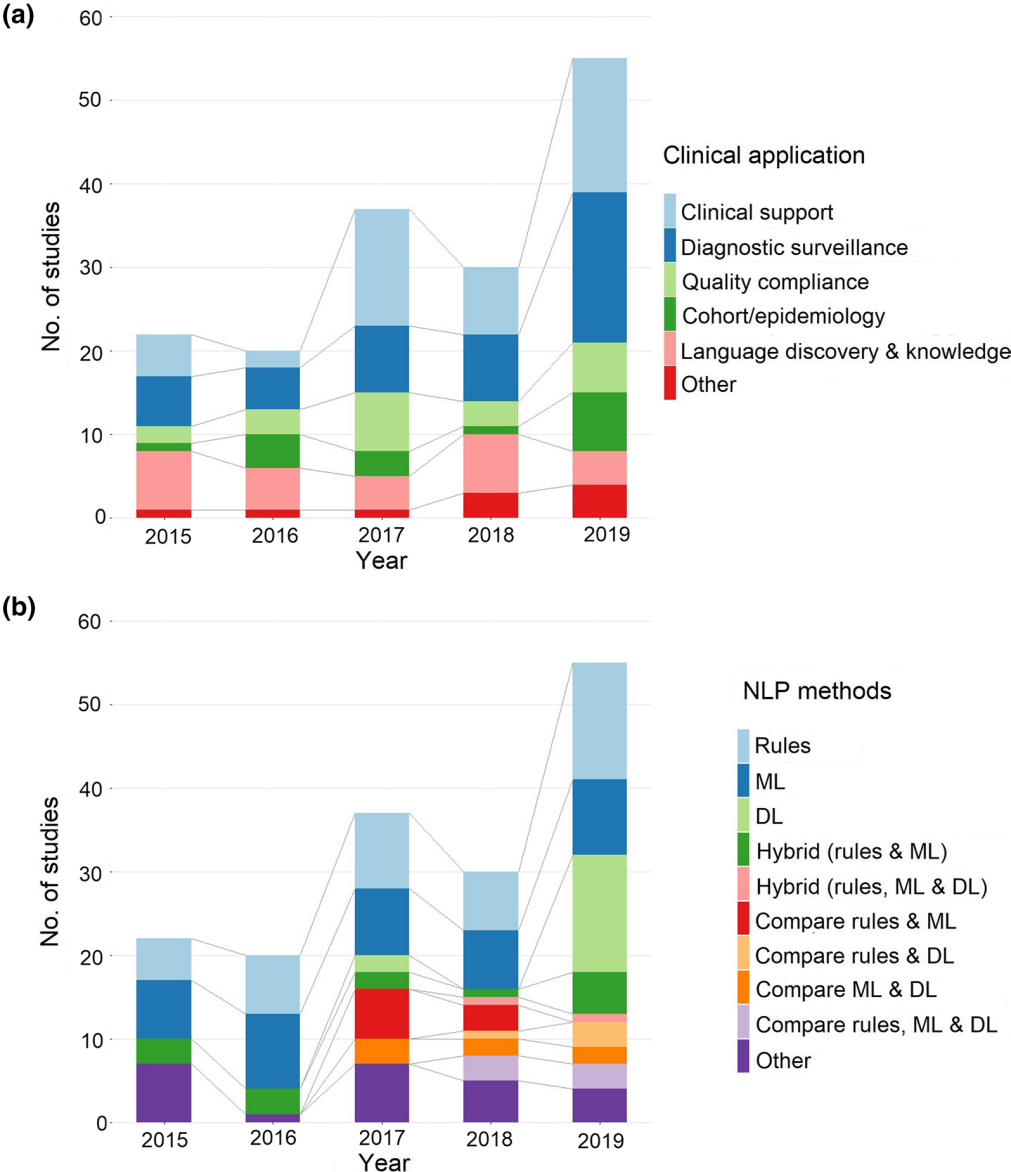
Distribution of studies by publication year and **a** clinical application, **b** NLP methods

Our search identified 4,836 publications of which 274 were potentially eligible. After full eligibility assessment, we included 164 studies that used NLP on radiology reports (Fig. 1). Figure 2 presents the number of studies identified by year and illustrates the breakdown of studies by both clinical application category and NLP method. The number of publications increased from 22 in 2015 to 55 in 2019 (up to October 2019). There were more studies using deep learning techniques more recently (Fig. 2). Table 1 attributes the studies to their clinical application categories and Table S1 (in Additional file 1) provides a detailed description of the study characteristics.

The most common clinical applications of these NLP studies were disease information or classification (28%) and diagnostic surveillance (27.4%), followed by language discovery and knowledge structure (16.5%), quality and compliance (12.2%) and then research (9.8%). Of the NLP methods used, rule-based alone (26%) and machine learning alone (24%) were most frequently applied. Deep learning methods alone were used in 16 studies (9.8%), rising from 0 in 2015 to 14 papers (25%) in 2019. More specifics of the NLP methods and clinical applications can be found in our accompanying informatics paper [13]. The majority (86%) of studies used English language radiology reports, with the other languages reported including Chinese, Spanish, German, French, Italian, Portuguese, Polish and Hebrew. The imaging modalities reported were mixed (28%), computerised tomography (23%), magnetic resonance imaging (9.8%), X-ray (4.9%), ultrasound (2.4%), mammography (3%), and other types (15%). The most frequent disease area was oncology (24%) and images of mixed anatomical regions were most frequent (26.2%), followed by thorax (19.5%) and head and neck (15.2%). The size of the datasets varied greatly between studies; eleven studies did not give data sizes; and others studies reported numbers of sentences, patients, or mixed data sources rather than numbers of reports. With these caveats, the median dataset size was 3,032 (IQR 875, 70 000).

### Reporting quality of included studies

**Fig. 3:**
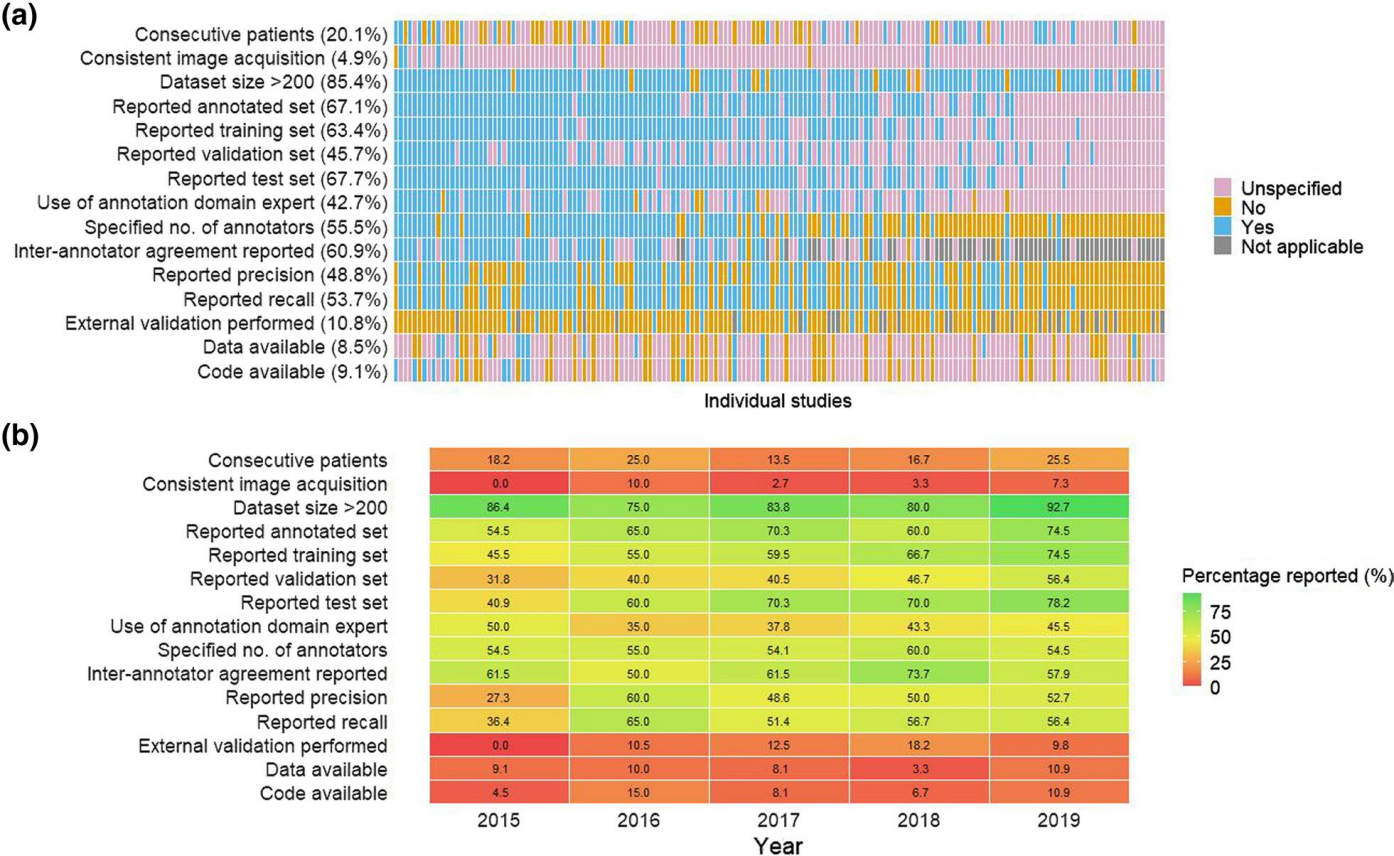
Quality of reporting in **a** individual studies and **b** between 2015 and 2019. *Legend*: **a** Studies are arranged by the total number of qualities reported in the study from left to right in descending order. **b** Numbers indicate the percentage of studies in each year of publication reporting the corresponding quality

Reporting of the pre-specified criteria varied across the included studies and years of publication (Fig. 3a, b). The median number of qualities achieved was 5. Consistent image acquisition was the most incompletely reported aspect of studies: 11 (6.7%) studies included information on the number and type of imaging machines used and just eight of the 11 studies specified that images were of consistent quality where various sites and imaging machines were used. Other criteria where incomplete reporting was particularly evident were reporting the results of external validation, only 15/139 (10.8%) studies; reporting of study data to make it available for external use, 14 (8.5%) studies; and the reporting of study code to make it available for external use, 15 (9.1%) studies.

The method for imaging reports sampling was also incompletely reported: 71 (43.3%) studies specified their sampling strategy, and only 33 (46.5%) of these studies sampled imaging reports consecutively. Most studies reported the size of their overall data set (93.3%) and 85.4% had a dataset size exceeding 200. However, the split of datasets in each study for training, validation, and test sets was reported only moderately well (63.4%, 45.7%, and 67.7% respectively). Annotated datasets were reported for 110 (67.1%) of the studies. Just under half of the studies (47.6%) reported the annotator expertise and 70 (42.7%) confirmed it was a domain expert. The number of annotators was specified in 91 (55.5%) studies and the inter-annotator agreement was reported for 67 (60.9%) of the 110 studies that used annotated data sets. We found that 80 (48.8%) and 88 (53.7%) studies, respectively, reported the performance metrics of precision and recall for their applications. There was no apparent improvement in reporting on visual inspection (Fig. 3b).

### Study performance

**Fig. 4:**
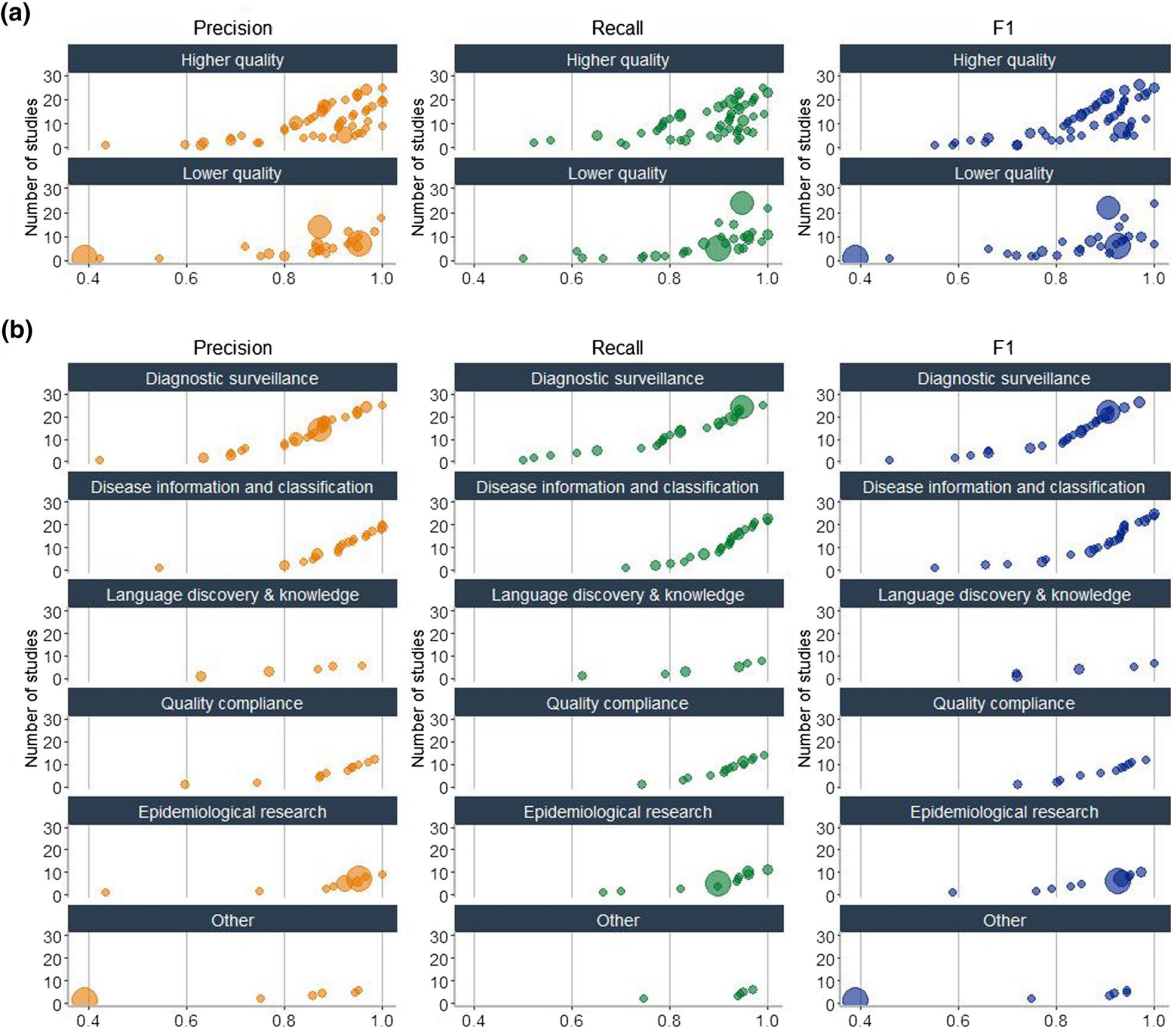
Precision, recall and F1 score by quality of reporting and clinical application category. *Legend*: NLP system performance reported as precision, recall and F1 score from included studies. Size of the bubbles represents the relative sizes of corpora in each graph. **a** Studies were categorised into high (> 5 qualities) and low (≤ 5 qualities) reporting quality based on the median number of qualities reported as the cut-off point. Reporting of F1 score was not a quality criterion. **b** Performance stratified by clinical application

In looking at study performance we also examined the 71 (43%) studies reporting F1 score. In studies reporting at least one of the performance measures (precision, recall and F1 score), there was no clear pattern of performance associated with quality of reporting or with stratification by clinical application (Fig. 4).

## Discussion

We conducted a systematic review of the quality of reporting of studies of NLP in radiology reports between 2015 and 2019. This review chronologically updated an existing review by Pons et al., although the focus of their review was on the clinical applications of NLP tools, NLP methods, and their performance, and did not assess quality of reporting. We found increased research output in the time period of our review, retrieving 164 relevant publications compared with 67 for the preceding review which searched for all publications indexed up to October, 2014. In our review, as anticipated, the use of deep learning methods had increased, but we found that rule-based and traditional machine classifiers were still widely used. The main clinical applications reported in papers remained broadly similar between the reviews, although we found more papers that did not specify any health-related purpose and we categorised these as: ‘Technical NLP’ and ‘Disease information and classification’. Pons et al. reported that many NLP tools remained at a ‘proof-of-concept’ stage and our study determined that this problem persists in the body of literature we retrieved.

The main focus of our work was on the reporting of clinical NLP studies and we found that this was generally poor (meaning under half of the included studies reported the criterion) for eight of our 15 pre-specified criteria. In particular, the three reproducibility criteria were met by only 15, 14 and 15 studies for external validation, availability of data and availability of code respectively. Although this is an expanding field, with a growth in publications, we found that reporting remained inconsistent and incomplete between 2015 and 2019.

Most studies reported dataset size. However, we found that more detailed information on data sampling was often omitted and that had implications for assessing bias in these studies. For example, not reporting whether imaging reports were sampled from consecutive patients and not detailing the demographics of study participants affected determination of selection bias and impacted on the generalisability of applications from one population to another. The dangers of utilising data from unrepresentative populations, particularly to train ML applications, has been stressed [19, 20] and considerations of equity and how models may vary across different settings have begun to be incorporated in existing guidance for ML [2]. The split of datasets between training, test and validation sets was also inadequately reported: 45.5%, 40.9% and 31.8% of studies published in 2015 reported these criteria respectively. However, these dataset criteria did appear to improve over time: 74.5%, 78.2% and 56.4% of studies published in 2019 reported these criteria respectively. Assessment of information bias was difficult because of the lack of details about comparable imaging machines and details of any annotation, including the number of annotators and whether they were domain experts.

Second, as recognised in ML [6] and DL [10] research, most NLP algorithms were ‘private’ and had not been replicated by their developers in other settings. It is therefore uncertain whether these tools are transferable between settings. External validation is difficult, because obtaining and accessing suitable alternate datasets on which to test NLP tools is not easy. There are few publicly available datasets and those which are available [21–24] may not be representative of the datasets researchers want to use or the populations for whom they are developing their tools. For example, clinical datasets available from the United States may not translate to another healthcare systems. External validation of clinical NLP tools is important to establish whether they can be adopted for more widespread use and clinical implementation.

Thirdly, external validation can be facilitated by the sharing of code and data to replicate research, but we found code was not available from many studies [25]. There are multiple institutional factors, some particular to healthcare data, which influence disclosure including privacy considerations, inconsistency in decision-making by regulatory bodies, liability concerns due to these technologies being viewed akin to medical devices, and lastly concerns over cybersecurity [26]. Additionally, NLP researchers may not have work capacity to support the use of their NLP systems when used externally. The development of bodies to facilitate health data research, such as Health Data Research UK (HDRUK) promises to address many of these factors [27], but they may remain a barrier for some time and, in the interim, encouraging direct collaboration between clinical NLP researchers working in similar areas may be the most efficient way to expedite external validation. There have been active steps taken by the NLP community towards improving reproducibility for ML in particular, including the development and implementation of reproducibility checklists specific to ML, and this shift in practice may spread to encompass other areas of the clinical NLP research community [28].

Fourth, specifying a clinical application is important to demonstrate that the tool has meaningful clinical relevance, and also because transferability of algorithms to different clinical tasks is not assured. Volmer et al. [2], recently proposed 20 questions concerning transparency, reproducibility, ethics, and effectiveness (TREE) for ML and AI research and their first question urges researchers from the inception of a project to stipulate the relevance of their work to healthcare and patients. This requirement is also born out in the CONSORT-AI reporting guidelines [4]. For our review, we generated six clinical application categories, extending Pons et al.’s existing framework [11] and disaggregating them into underlying subcategories, and we discovered that many studies did not specify a clinical application. Our study taxonomy may be useful for other researchers wishing to identify existing work to build on or to identify clinical areas with gaps that remain unaddressed. In addition, our inclusion of more disaggregated clinical application subcategories (Table 1) could potentially facilitate future work to collate these applications within ‘like’ categories to examine their performance in carrying out similar clinical tasks.

Lastly, we summarized the performance of all 164 studies and sought trends according to their quality of reporting and clinical application. However, no clear associations emerged. This is likely due to the heterogeneity within clinical NLP studies and their contextual nature. The best performing methods for a clinical NLP tool are likely determined by the intersection of multiple factors including clinical application, type of reports (including modality and indication), the specific information required (including rarity of conditions), the need for clinical input, the complexity of the NLP task being carried out, and the performance parameters required to be acceptable for clinical implementation and minimisation of harm.

The implications of our findings for practice are that, despite a large body of work and the potential advantages of NLP in clinical settings, advancing these tools to the stage of widespread implementation is hindered by poor standards of reporting, particularly relating to external validation and the sharing of NLP code and data. This reflects the situation reported for the sub-fields of ML and AI where systematic review identified that most studies failed to use or adhere to any existing reporting guidance [6, 10], and that data and code availability were lacking [10]. However, a move has begun to pursue transparency and replicability within AI, ML and DL research [2], which all clinical NLP should follow, including initiating the development of extended reporting guidelines [3, 4, 7]. Where no extended guidelines exist, we recommend that researchers follow guidelines specific to study type [29] and also consider reporting the 15 NLP specific criteria which we have sought in our review.

Our review was strengthened by the large number, and wide variety, of studies identified. However, the heterogeneity of this literature was also a limitation in that it precluded any meta-analysis of outcomes. Limitations of our review also included having developed our own quality assessment criteria, due to the lack of available tools in this field. We acknowledge that there may be additional criteria that could assist quality assessment either for specific types of NLP (such as hyperparameter selection for ML) or more generally; for example, including a description of computing infrastructure could also assist assessment of reproducibility and could be readily shared [18]. We also did not exclude any studies based on poor quality. However, we feel that this approach is fitting for a review where meta-analysis is not undertaken and where we focus on demonstrating the breadth of work and assessing reporting quality across the whole body of work. Utilising an automated search in Google Scholar may have impeded our search sensitivity, although it has been shown to have very comprehensive coverage [30]. Our clinical application categories were also developed through this review process and there was overlap for some studies where decisions had to be made by the reviewers as to their primary application. These decisions were naturally subjective and studies could be reassigned, however decisions were discussed and agreed by at least two reviewers.

## Conclusions and recommendations

Our systematic review of the use of NLP on radiology reports, for the period 2015–2019, found substantial growth in research activity, but no clear improvement in reporting of key data to allow reproducibility and replication. This impedes synthesis of this research field. In this paper we provide an overview of the current landscape and offer developments in both the categorisation of clinical applications for NLP on radiology reports and suggested criteria for inclusion in quality assessment of this research. This paper complements the limited guidance which has been published to date in relation to AI in radiology [9], clinical NLP [8], and ML within NLP [2–5] and we hope that our criteria can contribute to developments for formally agreed standards specific to clinical NLP.

## Supplementary Information

**Additional file 1**. Additional details of the automated search and more detailed characteristics.

## Data Availability

All data generated or analysed during this study are included in this published article and its companion paper [including their additional/supplementary information files] [13].

## Abbreviations

AI: Artificial Intelligence
CONSORT-AI: Consolidated Standards of Reporting Trials-Artificial Intelligence
CT: Computerised tomography
DL: Deep learning
HDRUK: Health Data Research UK
IQR: Interquartile Range
ML: Machine learning
MRI: Magnetic resonance imaging
NLP: Natural language processing
PRISMA: Preferred Reporting Items for Systematic Reviews and Meta-Analyses
TREE: Transparency, reproducibility, ethics, and effectiveness
