# Supplementary material for: The reporting quality of natural language processing studies: systematic review of studies of radiology reports
Source: BMC Med Imaging. 2021 Oct 2;21:142. doi: 10.1186/s12880-021-00671-8 (PMC8487512; doi:10.1186/s12880-021-00671-8)
Supplement: Supplementary file 1 — Additional file 1. Additional details of the automated search and more detailed characteristics. [file 12880_2021_671_MOESM1_ESM.docx]

# Additional File 1:

## Automated search and screening process

An automated search was carried out using Google scholar through Publish and Perish (a citation retrieval and analysis software programme). There was an initial pilot search conducted, but we found that the search terms were too specific and this limited the number of papers retrieved. We, therefore, extended our terms when we ran the search again. We also automated the addition of publication metadata. This involved:

1. Match the paper with its DOI via the Crossref API (<https://www.crossref.org/education/retrieve-metadata/rest-api/>)
2. If DOI matched, check Semantic Scholar (<https://www.semanticscholar.org/>) for metadata/abstract
3. If no DOI match and no abstract, search PubMed for abstract
4. Search arXiv (<https://arxiv.org/>) for a pre-print
5. If no PDF link, search Unpaywall (<https://library.lasalle.edu/unpaywall>) for available open access versions
6. If PDF but no separate abstract via Semantics Scholar/PubMed, extract abstract from the PDF

Automated screening was then applied to filter out irrelevant publications, and used the following exclusion criteria:

1. Document language is not English
2. Word 'patent' in title or URL
3. Year of publication out of range (<2015 – as our review aimed to update a previous review by Pons et al. (2016) which included literature up to October 2014)
4. The words 'review' or 'overview' in the title, 'this review' in the abstract
5. Image keywords in title or abstract with no NLP terminology in abstract
6. No radiology keywords in title or abstract
7. No NLP terminology in abstract

Lastly, we conducted a citation search. The citation search compiled a list of publications that cite Pons et al. (2016) review and the articles cited in their Pons’ review. To do this, we used a snowballing method to follow the forward citation branch for each publication in this list, i.e. finding every article that cites the publications in our list. The branching factor here is large, so we filtered at every stage and then automatically add the metadata.

**Supplementary Table S1: Characteristics of 164 included studies**

|  | 2015  (N=22) | 2016  (N=20) | 2017  (N=37) | 2018  (N=30) | 2019  (N=55) | Overall  (N=164) |
| --- | --- | --- | --- | --- | --- | --- |
|  | N (%) | N (%) | N (%) | N (%) | N (%) | N (%) |
| **Clinical application** |  |  |  |  |  |  |
| Diagnostic surveillance | 6 (27.3) | 5 (25.0) | 8 (21.6) | 8 (26.7) | 18 (32.7) | 45 (27.4) |
| Disease information and classification | 5 (22.7) | 3 (15.0) | 14 (37.8) | 8 (26.7) | 16 (29.1) | 46 (28.0) |
| Language discovery and knowledge structure | 7 (31.8) | 5 (25.0) | 4 (10.8) | 7 (23.3) | 4 (7.3) | 27 (16.5) |
| Quality and compliance | 2 (9.1) | 2 (10.0) | 7 (18.9) | 3 (10.0) | 6 (10.9) | 20 (12.2) |
| Research: Cohort and epidemiology | 1 (4.5) | 4 (20.0) | 3 (8.1) | 1 (3.3) | 7 (12.7) | 16 (9.8) |
| Technical NLP | 1 (4.5) | 1 (5.0) | 1 (2.7) | 3 (10) | 4 (7.3) | 10 (6.1) |
| **NLP methods** |  |  |  |  |  |  |
| Rule based only | 5 (23) | 7 (35) | 9 (24) | 7 (23) | 14 (25) | 42 (26) |
| Machine learning only | 7 (32) | 9 (45) | 8 (22) | 7 (23) | 9 (16) | 40 (24) |
| Deep learning only | 0 (0) | 0 (0) | 2 (5.4) | 0 (0) | 14 (25) | 16 (9.8) |
| Compare methods (in one paper) | 0 (0) | 0 (0) | 9 (24) | 9 (30) | 8 (15) | 26 (16) |
| Compare methods and create hybrid | 3 (14) | 3 (15) | 2 (5.4) | 2 (6.7) | 6 (11) | 16 (9.8) |
| NLP Bespoke Tool, Ontology only or Similarity Measure | 7 (32) | 1 (5) | 7 (19) | 5 (17) | 4 (7.3) | 24 (15) |
| **Total dataset size** |  |  |  |  |  |  |
| Median (IQR) | 7,500  (1,436, 132,488) | 1,600  (556,  21,906) | 3,060  (1,199, 13,982) | 2,478  (538, 101,896) | 3,046  (1,129, 188,571) | 3,032  (875, 70,000) |
| **Language of reports** |  |  |  |  |  |  |
| English | 18 (81.8) | 16 (80.0) | 33 (89.2) | 23 (76.7) | 51 (92.7) | 141 (86.0) |
| Chinese | 0 (0) | 1 (5) | 0 (0) | 2 (6.7) | 2 (3.6) | 5 (3) |
| Portuguese | 0 (0) | 1 (5) | 0 (0) | 0 (0) | 0 (0) | 1 (0.6) |
| German | 1 (4.5) | 0 (0) | 0 (0) | 2 (6.7) | 0 (0) | 3 (1.8) |
| Italian | 1 (4.5) | 0 (0) | 1 (2.7) | 0 (0) | 0 (0) | 2 (1.2) |
| Polish | 0 (0) | 0 (0) | 0 (0) | 1 (3.3) | 0 (0) | 1 (0.6) |
| Spanish | 1 (4.5) | 1 (5) | 0 (0) | 2 (6.7) | 0 (0) | 4 (2.4) |
| French | 0 (0) | 1 (5) | 1 (2.7) | 0 (0) | 0 (0) | 2 (1.2) |
| Hebrew | 0 (0) | 0 (0) | 0 (0) | 0 (0) | 1 (1.8) | 1 (0.6) |
| Unspecified | 1 (4.5) | 0 (0) | 2 (5.4) | 0 (0) | 1 (1.8) | 4 (2.4) |
| **Imaging modality** |  |  |  |  |  |  |
| Computerised tomography | 4 (18) | 3 (15) | 9 (24) | 6 (20) | 16 (29) | 38 (23) |
| Magnetic resonance imaging | 0 (0) | 1 (5) | 5 (14) | 2 (6.7) | 8 (15) | 16 (9.8) |
| Ultrasound | 0 (0) | 2 (10) | 1 (2.7) | 1 (3.3) | 0 (0) | 4 (2.4) |
| X-ray | 2 (9.1) | 1 (5) | 2 (5.4) | 1 (3.3) | 2 (3.6) | 8 (4.9) |
| Mammogram | 1 (4.5) | 1 (5) | 1 (2.7) | 2 (6.7) | 0 (0) | 5 (3) |
| Mixed | 9 (41) | 5 (25) | 12 (32) | 5 (17) | 15 (27) | 46 (28) |
| Others | 5 (23) | 1 (5) | 4 (11) | 6 (20) | 8 (15) | 24 (15) |
| Unspecified | 1 (4.5) | 6 (30) | 3 (8.1) | 7 (23) | 6 (11) | 23 (14) |
| **Anatomical region** |  |  |  |  |  |  |
| Head/neck | 0 (0) | 3 (15) | 6 (16.2) | 5 (16.7) | 11 (20) | 25 (15.2) |
| Thorax | 5 (22.7) | 1 (5) | 9 (24.3) | 5 (16.7) | 12 (21.8) | 32 (19.5) |
| Breast | 2 (9.1) | 4 (20) | 2 (5.4) | 4 (13.3) | 3 (5.5) | 15 (9.1) |
| Abdomen | 1 (4.5) | 3 (15) | 5 (13.5) | 1 (3.3) | 5 (9.1) | 15 (9.1) |
| Spine | 0 (0) | 1 (5) | 1 (2.7) | 1 (3.3) | 2 (3.6) | 5 (3) |
| Extremities | 1 (4.5) | 1 (5) | 3 (8.1) | 3 (10) | 1 (1.8) | 9 (5.5) |
| Mixed | 8 (36.4) | 4 (20) | 9 (24.3) | 8 (26.7) | 14 (25.5) | 43 (26.2) |
| Other | 0 (0) | 0 (0) | 0 (0) | 0 (0) | 1 (1.8) | 1 (0.6) |
| Unspecified | 5 (22.7) | 3 (15) | 2 (5.4) | 3 (10) | 6 (10.9) | 19 (11.6) |
| **Disease of interest** |  |  |  |  |  |  |
| Oncology | 6 (27) | 6 (30) | 9 (24) | 7 (23) | 11 (20) | 39 (24) |
| Cardiovascular | 0 (0) | 1 (5) | 3 (8.1) | 1 (3.3) | 1 (1.8) | 6 (3.7) |
| Cerebrovascular | 0 (0) | 0 (0) | 2 (5.4) | 2 (6.7) | 9 (16) | 13 (7.9) |
| Respiratory | 1 (4.5) | 1 (5) | 2 (5.4) | 2 (6.7) | 4 (7.3) | 10 (6.1) |
| Musculoskeletal | 0 (0) | 1 (5) | 3 (8.1) | 3 (10) | 3 (5.5) | 10 (6.1) |
| Gastrointestinal | 0 (0) | 0 (0) | 2 (5.4) | 1 (3.3) | 0 (0) | 3 (1.8) |
| Genitourinary | 0 (0) | 0 (0) | 0 (0) | 0 (0) | 1 (1.8) | 1 (0.6) |
| Hepatobiliary | 0 (0) | 0 (0) | 1 (2.7) | 0 (0) | 1 (1.8) | 2 (1.2) |
| Trauma | 0 (0) | 3 (15) | 0 (0) | 1 (3.3) | 3 (5.5) | 7 (4.3) |
| Mixed | 1 (4.5) | 1 (5) | 5 (14) | 3 (10) | 10 (18) | 20 (12) |
| Other | 3 (14) | 2 (10) | 5 (14) | 2 (6.7) | 1 (1.8) | 13 (7.9) |
| Unspecified | 11 (50) | 5 (25) | 5 (14) | 8 (27) | 11 (20) | 40 (24.4) |

**List of 164 review papers:**

1 Peng Y, Yan K, Sandfort V, *et al.* A self-attention based deep learning method for lesion attribute detection from CT reports. In: *2019 IEEE International Conference on Healthcare Informatics (ICHI)*. Xi’an, China: : IEEE Computer Society 2019. 1–5. doi:10.1109/ICHI.2019.8904668

2  Bozkurt S, Alkim E, Banerjee I, *et al.* Automated Detection of Measurements and Their Descriptors in Radiology Reports Using a Hybrid Natural Language Processing Algorithm. *J Digit Imaging* 2019;**32**:544–53. doi:10.1007/s10278-019-00237-9

3  Hassanpour S, Bay G, Langlotz CP. Characterization of Change and Significance for Clinical Findings in Radiology Reports Through Natural Language Processing. *J Digit Imaging* 2017;**30**:314–22. doi:10.1007/s10278-016-9931-8

4  Kehl KL, Elmarakeby H, Nishino M, *et al.* Assessment of Deep Natural Language Processing in Ascertaining Oncologic Outcomes From Radiology Reports. *JAMA Oncol* 2019;**5**:1421–9. doi:10.1001/jamaoncol.2019.1800

5  Chen P-H, Zafar H, Galperin-Aizenberg M, *et al.* Integrating Natural Language Processing and Machine Learning Algorithms to Categorize Oncologic Response in Radiology Reports. *J Digit Imaging* 2018;**31**:178–84. doi:10.1007/s10278-017-0027-x

6  Cotik V, Rodríguez H, Vivaldi J. Spanish Named Entity Recognition in the Biomedical Domain. In: Lossio-Ventura JA, Muñante D, Alatrista-Salas H, eds. *Information Management and Big Data*. Lima, Peru: : Springer International Publishing 2018. 233–48. doi:10.1007/978-3-030-11680-4_23

7  Sevenster M, Buurman J, Liu P, *et al.* Natural Language Processing Techniques for Extracting and Categorizing Finding Measurements in Narrative Radiology Reports. *Appl Clin Inform* 2015;**06**:600–10. doi:10.4338/ACI-2014-11-RA-0110

8  Sevenster M, Bozeman J, Cowhy A, *et al.* A natural language processing pipeline for pairing measurements uniquely across free-text CT reports. *J Biomed Inform* 2015;**53**:36–48. doi:10.1016/j.jbi.2014.08.015

9  Oberkampf H, Zillner S, Overton JA, *et al.* Semantic representation of reported measurements in radiology. *BMC Med Inform Decis Mak* 2016;**16**:5. doi:10.1186/s12911-016-0248-9

10  Liu Y, Zhu L-N, Liu Q, *et al.* Automatic extraction of imaging observation and assessment categories from breast magnetic resonance imaging reports with natural language processing. *Chin Med J (Engl)* 2019;**132**:1673–80. doi:10.1097/CM9.0000000000000301

11  Gupta A, Banerjee I, Rubin DL. Automatic information extraction from unstructured mammography reports using distributed semantics. *J Biomed Inform* 2018;**78**:78–86. doi:10.1016/j.jbi.2017.12.016

12  Castro SM, Tseytlin E, Medvedeva O, *et al.* Automated annotation and classification of BI-RADS assessment from radiology reports. *J Biomed Inform* 2017;**69**:177–87. doi:10.1016/j.jbi.2017.04.011

13  Short RG, Bralich J, Bogaty D, *et al.* Comprehensive Word-Level Classification of Screening Mammography Reports Using a Neural Network Sequence Labeling Approach. *J Digit Imaging* 2019;**32**:685–92. doi:10.1007/s10278-018-0141-4

14  Lacson R, Goodrich ME, Harris K, *et al.* Assessing Inaccuracies in Automated Information Extraction of Breast Imaging Findings. *J Digit Imaging* 2017;**30**:228–33. doi:10.1007/s10278-016-9927-4

15  Lacson R, Harris K, Brawarsky P, *et al.* Evaluation of an Automated Information Extraction Tool for Imaging Data Elements to Populate a Breast Cancer Screening Registry. *J Digit Imaging* 2015;**28**:567–75. doi:10.1007/s10278-014-9762-4

16  Yim W, Kwan SW, Yetisgen M. Tumor reference resolution and characteristic extraction in radiology reports for liver cancer stage prediction. *J Biomed Inform* 2016;**64**:179–91. doi:10.1016/j.jbi.2016.10.005

17  Yim W, Kwan SW, Yetisgen M. Classifying tumor event attributes in radiology reports. *J Assoc Inf Sci Technol* 2017;**68**:2662–74. doi:10.1002/asi.23937

18  Yim W, Denman T, Kwan SW, *et al.* Tumor information extraction in radiology reports for hepatocellular carcinoma patients. *AMIA Summits Transl Sci Proc* 2016;**2016**:455–64.

19  Pruitt P, Naidech A, Van Ornam J, *et al.* A natural language processing algorithm to extract characteristics of subdural hematoma from head CT reports. *Emerg Radiol* 2019;**26**:301–6. doi:10.1007/s10140-019-01673-4

20  Farjah F, Halgrim S, Buist DSM, *et al.* An Automated Method for Identifying Individuals with a Lung Nodule Can Be Feasibly Implemented Across Health Systems. *eGEMs* 2016;**4**:1254. doi:10.13063/2327-9214.1254

21  Karunakaran B, Misra D, Marshall K, *et al.* Closing the loop — Finding lung cancer patients using NLP. In: *2017 IEEE International Conference on Big Data (Big Data)*. Boston, MA: : IEEE 2017. 2452–61. doi:10.1109/BigData.2017.8258203

22  Tan WK, Hassanpour S, Heagerty PJ, *et al.* Comparison of Natural Language Processing Rules-based and Machine-learning Systems to Identify Lumbar Spine Imaging Findings Related to Low Back Pain. *Acad Radiol* 2018;**25**:1422–32. doi:10.1016/j.acra.2018.03.008

23  Trivedi G, Hong C, Dadashzadeh ER, *et al.* Identifying incidental findings from radiology reports of trauma patients: An evaluation of automated feature representation methods. *Int J Med Inf* 2019;**129**:81–7. doi:10.1016/j.ijmedinf.2019.05.021

24  Fu S, Leung LY, Wang Y, *et al.* Natural Language Processing for the Identification of Silent Brain Infarcts From Neuroimaging Reports. *JMIR Med Inform* 2019;**7**:e12109. doi:10.2196/12109

25  Ananda-Rajah MR, Bergmeir C, Petitjean F, *et al.* Toward Electronic Surveillance of Invasive Mold Diseases in Hematology-Oncology Patients: An Expert System Combining Natural Language Processing of Chest Computed Tomography Reports, Microbiology, and Antifungal Drug Data. *JCO Clin Cancer Inform* 2017;**1**:1–10. doi:10.1200/CCI.17.00011

26  Wang Y, Mehrabi S, Sohn S, *et al.* Natural language processing of radiology reports for identification of skeletal site-specific fractures. *BMC Med Inform Decis Mak* 2019;**19**:73. doi:10.1186/s12911-019-0780-5

27  Baggio D, Peel T, Peleg AY, *et al.* Closing the gap in surveillance and audit of invasive mold diseases for antifungal stewardship using machine learning. *J Clin Med* 2019;**8**:1390. doi:10.3390/jcm8091390

28  Afshar M, Joyce C, Oakey A, *et al.* A Computable Phenotype for Acute Respiratory Distress Syndrome Using Natural Language Processing and Machine Learning. *AMIA Annu Symp Proc* 2018;**2018**:157–65.

29  Chapman AB, Mowery DL, Swords DS, *et al.* Detecting Evidence of Intra-abdominal Surgical Site Infections from Radiology Reports Using Natural Language Processing. *AMIA Annu Symp Proc* 2018;**2017**:515–24.

30  Tian Z, Sun S, Eguale T, *et al.* Automated Extraction of VTE Events From Narrative Radiology Reports in Electronic Health Records. *Med Care* 2017;**55**:e73–80. doi:10.1097/MLR.0000000000000346

31  Annarumma M, Withey SJ, Bakewell RJ, *et al.* Automated Triaging of Adult Chest Radiographs with Deep Artificial  Neural Networks. *Radiology* 2019;**291**:196–202. doi:10.1148/radiol.2018180921

32  Singh M, Murthy A, Singh S. Prioritization of Free-Text Clinical Documents: A Novel Use of a Bayesian Classifier. *JMIR Med Inform* 2015;**3**:e17. doi:10.2196/medinform.3793

33  Meng X, Ganoe CH, Sieberg RT, *et al.* Assisting radiologists with reporting urgent findings to referring physicians: A machine learning approach to identify cases for prompt communication. *J Biomed Inform* 2019;**93**:103169. doi:10.1016/j.jbi.2019.103169

34  Ratner A, Hancock B, Dunnmon J, *et al.* Snorkel MeTaL: Weak Supervision for Multi-Task Learning. In: *Proceedings of the Second Workshop on Data Management for End-To-End Machine Learning*. Houston, TX, USA: : ACM 2018. 1–4. doi:10.1145/3209889.3209898

35  Gupta EK, Thammasudjarit R, Thakkinstian A. A Hybrid Engine for Clinical Information Extraction from Radiology Reports. In: *2019 16th International Joint Conference on Computer Science and Software Engineering (JCSSE)*. Chonburi, Thailand: : IEEE 2019. 293–7. doi:10.1109/JCSSE.2019.8864178

36  Tahmasebi AM, Zhu H, Mankovich G, *et al.* Automatic Normalization of Anatomical Phrases in Radiology Reports Using Unsupervised Learning. *J Digit Imaging* 2019;**32**:6–18. doi:10.1007/s10278-018-0116-5

37  Khor RC, Nguyen A, O’Dwyer J, *et al.* Extracting tumour prognostic factors from a diverse electronic record dataset in genito-urinary oncology. *Int J Med Inf* 2019;**121**:53–7. doi:10.1016/j.ijmedinf.2018.10.008

38  Zhu H, Paschalidis ICh, Hall C, *et al.* Context-Driven Concept Annotation in Radiology Reports: Anatomical Phrase Labeling. *AMIA Summits Transl Sci Proc* 2019;**2019**:232–41.

39  Yang H, Li L, Yang R, *et al.* Towards Automated Knowledge Discovery of Hepatocellular Carcinoma: Extract Patient Information from Chinese Clinical Reports. In: *Proceedings of the 2nd International Conference on Medical and Health Informatics*. New York, NY, USA: : ACM 2018. 111–116. doi:10.1145/3239438.3239445

40  Gerevini AE, Lavelli A, Maffi A, *et al.* Automatic classification of radiological reports for clinical care. *Artif Intell Med* 2018;**91**:72–81. doi:10.1016/j.artmed.2018.05.006

41  Martinez D, Ananda-Rajah MR, Suominen H, *et al.* Automatic detection of patients with invasive fungal disease from free-text computed tomography (CT) scans. *J Biomed Inform* 2015;**53**:251–60. doi:10.1016/j.jbi.2014.11.009

42  Lou R, Lalevic D, Chambers C, *et al.* Automated Detection of Radiology Reports that Require Follow-up Imaging Using Natural Language Processing Feature Engineering and Machine Learning Classification. *J Digit Imaging* 2020;**33**:131–6. doi:10.1007/s10278-019-00271-7

43  Lau W, Payne TH, Uzuner O, *et al.* Extraction and Analysis of Clinically Important Follow-up Recommendations in a Large Radiology Dataset. *ArXiv [Cs.CL]* 2019. http://arxiv.org/abs/1905.05877 (accessed 30 Oct 2020).

44  Cochon LR, Kapoor N, Carrodeguas E, *et al.* Variation in Follow-up Imaging Recommendations in Radiology Reports: Patient, Modality, and Radiologist Predictors. *Radiology* 2019;**291**:700–7. doi:10.1148/radiol.2019182826

45  Carrodeguas E, Lacson R, Swanson W, *et al.* Use of Machine Learning to Identify Follow-Up Recommendations in Radiology Reports. *J Am Coll Radiol JACR* 2019;**16**:336–43. doi:10.1016/j.jacr.2018.10.020

46  Jnawali K, Arbabshirani MR, Ulloa AE, *et al.* Automatic Classification of Radiological Report for Intracranial Hemorrhage. In: *2019 IEEE 13th International Conference on Semantic Computing (ICSC)*. Newport Beach, CA, USA: : IEEE 2019. 187–90. doi:10.1109/ICOSC.2019.8665578

47  Banerjee I, Madhavan S, Goldman RE, *et al.* Intelligent Word Embeddings of Free-Text Radiology Reports. *AMIA Annu Symp Proc* 2017;:411–20.

48  Kłos M, Żyłkowski J, Spinczyk D. Automatic Classification of Text Documents Presenting Radiology Examinations. In: Pietka E, Badura P, Kawa J, *et al.*, eds. *Proceedings 6th International Conference Information Technology in Biomedicine(ITIB’2018)*. Kamień Śląski, Poland: : Springer International Publishing 2018. 495–505. doi:10.1007/978-3-319-91211-0_43

49  Deshmukh N, Gumustop S, Gauriau R, *et al.* Semi-Supervised Natural Language Approach for Fine-Grained Classification of Medical Reports. *ArXiv[Cs.LG]*2019. http://arxiv.org/abs/1910.13573 (accessed 30 Oct 2020).

50  Kim C, Zhu V, Obeid J, *et al.* Natural language processing and machine learning algorithm to identify brain MRI reports with acute ischemic stroke. *PLOS ONE* 2019;**14**:e0212778. doi:10.1371/journal.pone.0212778

51  Garg R, Oh E, Naidech A, *et al.* Automating Ischemic Stroke Subtype Classification Using Machine Learning and Natural Language Processing. *J Stroke Cerebrovasc Dis* 2019;**28**:2045–51. doi:10.1016/j.jstrokecerebrovasdis.2019.02.004

52  Shin B, Chokshi FH, Lee T, *et al.* Classification of radiology reports using neural attention models. In: *2017 International Joint Conference on Neural Networks (IJCNN)*. Anchorage, AK: : IEEE 2017. 4363–70. doi:10.1109/IJCNN.2017.7966408

53  Wheater E, Mair G, Sudlow C, *et al.* A validated natural language processing algorithm for brain imaging phenotypes from radiology reports in UK electronic health records. *BMC Med Inform Decis Mak* 2019;**19**:184. doi:10.1186/s12911-019-0908-7

54  Gorinski PJ, Wu H, Grover C, *et al.* Named Entity Recognition for Electronic Health Records: A Comparison of Rule-based and Machine Learning Approaches. *ArXiv[Cs.CL] 2*019. http://arxiv.org/abs/1903.03985 (accessed 30 Oct 2020).

55  Alex B, Grover C, Tobin R, *et al.* Text mining brain imaging reports. *J Biomed Semant* 2019;**10**:23. doi:10.1186/s13326-019-0211-7

56  Bozkurt S, Gimenez F, Burnside ES, *et al.* Using automatically extracted information from mammography reports for decision-support. *J Biomed Inform* 2016;**62**:224–31. doi:10.1016/j.jbi.2016.07.001

57  Patel TA, Puppala M, Ogunti RO, *et al.* Correlating mammographic and pathologic findings in clinical decision support using natural language processing and data mining methods. *Cancer* 2017;**123**:114–21. doi:10.1002/cncr.30245

58  Banerjee I, Bozkurt S, Alkim E, *et al.* Automatic inference of BI-RADS final assessment categories from narrative mammography report findings. *J Biomed Inform* 2019;**92**:103137. doi:10.1016/j.jbi.2019.103137

59  Miao S, Xu T, Wu Y, *et al.* Extraction of BI-RADS findings from breast ultrasound reports in Chinese using deep learning approaches. *Int J Med Inf* 2018;**119**:17–21. doi:10.1016/j.ijmedinf.2018.08.009

60  Dunne RM, Ip IK, Abbett S, *et al.* Effect of Evidence-based Clinical Decision Support on the Use and Yield of CT Pulmonary Angiographic Imaging in Hospitalized Patients. *Radiology* 2015;**276**:167–74. doi:10.1148/radiol.15141208

61  Banerjee I, Ling Y, Chen MC, *et al.* Comparative effectiveness of convolutional neural network (CNN) and recurrent neural network (RNN) architectures for radiology text report classification. *Artif Intell Med* 2019;**97**:79–88. doi:10.1016/j.artmed.2018.11.004

62  Chen MC, Ball RL, Yang L, *et al.* Deep Learning to Classify Radiology Free-Text Reports. *Radiology* 2017;**286**:845–52. doi:10.1148/radiol.2017171115

63  Meystre S, Gouripeddi R, Tieder J, *et al.* Enhancing Comparative Effectiveness Research With Automated Pediatric Pneumonia Detection in a Multi-Institutional Clinical Repository: A PHIS+ Pilot Study. *J Med Internet Res* 2017;**19**:e162. doi:10.2196/jmir.6887

64  Beyer SE, McKee BJ, Regis SM, *et al.* Automatic Lung-RADS^TM^ classification with a natural language processing system. *J Thorac Dis* 2017;**9**:3114–22. doi:10.21037/jtd.2017.08.13

65  Banerjee I, Bozkurt S, Caswell-Jin JL, *et al.* Natural Language Processing Approaches to Detect the Timeline of Metastatic Recurrence of Breast Cancer. *JCO Clin Cancer Inform* 2019;:1–12. doi:10.1200/CCI.19.00034

66  Chen L, Song L, Shao Y, *et al.* Using natural language processing to extract clinically useful information from Chinese electronic medical records. *Int J Med Inf* 2019;**124**:6–12. doi:10.1016/j.ijmedinf.2019.01.004

67  Rafeh R, Ahmadi M. A New Approach for Classifying Radiology Reports. *J Med Imaging Health Inform* 2015;**5**:257–63. doi:10.1166/jmihi.2015.1383

68  Liventsev V, Fedulova I, Dylov D. Deep Text Prior: Weakly Supervised Learning for Assertion Classification. In: Tetko IV, Kůrková V, Karpov P, *et al.*, eds. *Artificial Neural Networks and Machine Learning – ICANN 2019: Workshop and Special Sessions*. Munich, Germany: : Springer International Publishing 2019. 243–57. doi:10.1007/978-3-030-30493-5_26

69  Kim Y, Garvin JH, Goldstein MK, *et al.* Extraction of left ventricular ejection fraction information from various types of clinical reports. *J Biomed Inform* 2017;**67**:42–8. doi:10.1016/j.jbi.2017.01.017

70  Patterson OV, Freiberg MS, Skanderson M, *et al.* Unlocking echocardiogram measurements for heart disease research through natural language processing. *BMC Cardiovasc Disord* 2017;**17**:151. doi:10.1186/s12872-017-0580-8

71  Dantes RB, Zheng S, Lu JJ, *et al.* Improved Identification of Venous Thromboembolism From Electronic Medical Records Using a Novel Information Extraction Software Platform. *Med Care* 2018;**56**:e54. doi:10.1097/MLR.0000000000000831

72  Haan RR de, Visser JBR, Pons E, *et al.* Patient-specific workup of adrenal incidentalomas. *Eur J Radiol Open* 2017;**4**:108–14. doi:10.1016/j.ejro.2017.08.002

73  Collier N, Oellrich A, Groza T. Concept selection for phenotypes and diseases using learn to rank. *J Biomed Semant* 2015;**6**:24. doi:10.1186/s13326-015-0019-z

74  Senders JT, Karhade AV, Cote DJ, *et al.* Natural Language Processing for Automated Quantification of Brain Metastases Reported in Free-Text Radiology Reports. *JCO Clin Cancer Inform* 2019;:1–9. doi:10.1200/CCI.18.00138

75  Zech J, Pain M, Titano J, *et al.* Natural Language–based Machine Learning Models for the                     Annotation of Clinical Radiology Reports. *Radiology* 2018;**287**:570–80. doi:10.1148/radiol.2018171093

76  Kayi ES, Yadav K, Chamberlain JM, *et al.* Topic Modeling for Classification of Clinical Reports. *ArXiv[Cs.CL]* 2017. http://arxiv.org/abs/1706.06177 (accessed 30 Oct 2020).

77  Yim W, Kwan SW, Johnson G, *et al.* Classification of hepatocellular carcinoma stages from free-text clinical and radiology reports. *AMIA Annu Symp Proc* 2018;**2017**:1858–67.

78  Grundmeier RW, Masino AJ, Casper TC, *et al.* Identification of Long Bone Fractures in Radiology Reports Using Natural Language Processing to support Healthcare Quality Improvement. *Appl Clin Inform* 2016;**07**:1051–68. doi:10.4338/ACI-2016-08-RA-0129

79  Lee C, Kim Y, Kim YS, *et al.* Automatic Disease Annotation From Radiology Reports Using Artificial Intelligence Implemented by a Recurrent Neural Network. *Am J Roentgenol* 2019;**212**:734–40. doi:10.2214/AJR.18.19869

80  Hassanzadeh H, Kholghi M, Nguyen A, *et al.* Clinical Document Classification Using Labeled and Unlabeled Data Across Hospitals. *AMIA Annu Symp Proc* 2018;**2018**:545–54.

81  Swartz J, Koziatek C, Theobald J, *et al.* Creation of a simple natural language processing tool to support an imaging utilization quality dashboard. *Int J Med Inf* 2017;**101**:93–9. doi:10.1016/j.ijmedinf.2017.02.011

82  Fiebeck J, Laser H, Winther HB, *et al.* Leaving No Stone Unturned: Using Machine Learning Based Approaches for Information Extraction from Full Texts of a Research Data Warehouse. In: Auer S, Vidal M-E, eds. *13th International Conference Data Integration in the Life Sciences (DILS 2018)*. Hannover, Germany: : Springer International Publishing 2018. 50–8. doi:10.1007/978-3-030-06016-9_5

83  Gálvez JA, Pappas JM, Ahumada L, *et al.* The use of natural language processing on pediatric diagnostic radiology reports in the electronic health record to identify deep venous thrombosis in children. *J Thromb Thrombolysis* 2017;**44**:281–90. doi:10.1007/s11239-017-1532-y

84  Hassanzadeh H, Nguyen A, Karimi S, *et al.* Transferability of artificial neural networks for clinical document classification across hospitals: A case study on abnormality detection from radiology reports. *J Biomed Inform* 2018;**85**:68–79. doi:10.1016/j.jbi.2018.07.017

85  Kavuluru R, Rios A, Lu Y. An empirical evaluation of supervised learning approaches in assigning diagnosis codes to electronic medical records. *Artif Intell Med* 2015;**65**:155–66. doi:10.1016/j.artmed.2015.04.007

86  Karimi S, Dai X, Hassanzadeh H, *et al.* Automatic Diagnosis Coding of Radiology Reports: A Comparison of Deep Learning and Conventional Classification Methods. In: *BioNLP 2017*. Vancouver, Canada: : Association for Computational Linguistics 2017. 328–332. doi:10.18653/v1/W17-2342

87  Kocbek S, Cavedon L, Martinez D, *et al.* Evaluating classification power of linked admission data sources with text mining. In: *Proceedings of the Scientific Stream at Big Data in Health Analytics 2015 (BigData 2015)*. Swissotel Sydney, Sydney, Australia: : Ruzica Piskac 2015. 1–7.http://ceur-ws.org/Vol-1468/

88  Krishnan GS, Kamath S. S. Ontology-driven Text Feature Modeling for Disease Prediction using Unstructured Radiological Notes. *Comput Sist* 2019;**23**. doi:10.13053/cys-23-3-3238

89  Kocbek S, Cavedon L, Martinez D, *et al.* Text mining electronic hospital records to automatically classify admissions against disease: Measuring the impact of linking data sources. *J Biomed Inform* 2016;**64**:158–67. doi:10.1016/j.jbi.2016.10.008

90  Pandya J, Ganda K, Ridley L, *et al.* Identification of Patients with Osteoporotic Vertebral Fractures via Simple Text Search of Routine Radiology Reports. *Calcif Tissue Int* 2019;**105**:156–60. doi:10.1007/s00223-019-00557-6

91  Qenam B, Kim TY, Carroll MJ, *et al.* Text Simplification Using Consumer Health Vocabulary to Generate Patient-Centered Radiology Reporting: Translation and Evaluation. *J Med Internet Res* 2017;**19**:e417. doi:10.2196/jmir.8536

92  Lafourcade M, Ramadier L. Radiological text simplification using a general knowledge base. In: *18th International Conference on Computational Linguistics and Intelligent Text Processing (CICLing 2017)*. Budapest, Hungary 2017. doi:https://doi.org/10.1007/978-3-319-77116-8_46

93  Hong Y, Zhang J. Investigation of Terminology Coverage in Radiology Reporting Templates and Free‐text Reports. *Int J Knowl Content Dev Technol* 2015;**5**:5–14. doi:10.5865/IJKCT.2015.5.1.005

94  Comelli A, Agnello L, Vitabile S. An ontology-based retrieval system for mammographic reports. In: *2015 IEEE Symposium on Computers and Communication (ISCC)*. Larnaca: : IEEE 2015. 1001–6. doi:10.1109/ISCC.2015.7405644

95  Cotik Viviana, Filippo D, Castano J. An Approach for Automatic Classification of Radiology Reports in Spanish. *Stud Health Technol Inform* 2015;**216**:634–8.

96  Johnson E, Baughman WC, Ozsoyoglu G. A method for imputation of semantic class in diagnostic radiology text. In: *2015 IEEE International Conference on Bioinformatics and Biomedicine (BIBM)*. Washington, DC: : IEEE 2015. 750–5. doi:10.1109/BIBM.2015.7359780

97  Mujjiga S, Krishna V, Chakravarthi K, *et al.* Identifying Semantics in Clinical Reports Using Neural Machine Translation. *Proc AAAI Conf Artif Intell* 2019;**33**:9552–7. doi:10.1609/aaai.v33i01.33019552

98  Lafourcade M, Ramadier L. Semantic RelationExtraction with Semantic Patterns: Experiment on Radiology Report. In: *Proceedings of the Tenth International Conference on Language Resources and Evaluation (LREC 2016)*. Portorož, Slovenia: : European Language Resources Association (ELRA) 2016. https://hal.archives-ouvertes.fr/hal-01382320

99  Martin-Carreras T, Kahn CE. Coverage and Readability of Information Resources to Help Patients Understand Radiology Reports. *J Am Coll Radiol* 2018;**15**:1681–6. doi:10.1016/j.jacr.2017.11.019

100  Hassanpour S, Langlotz CP. Unsupervised Topic Modeling in a Large Free Text Radiology Report Repository. *J Digit Imaging* 2016;**29**:59–62. doi:10.1007/s10278-015-9823-3

101  Kovacs MD, Mesterhazy J, Avrin D, *et al.* Correlate: A PACS- and EHR-integrated Tool Leveraging Natural Language Processing to Provide Automated Clinical Follow-up. *RadioGraphics* 2017;**37**:1451–60. doi:10.1148/rg.2017160195

102  Lalithsena S, Tari L, Reden A von, *et al.* Feedback-Driven Radiology Exam Report Retrieval with Semantics. In: *Proceedings of the 2015 International Conference on Healthcare Informatics*. Dallas, Texas: : IEEE Computer Society 2015. 233–42. doi:10.1109/ICHI.2015.35

103  Zhao Y, Fesharaki NJ, Liu H, *et al.* Using data-driven sublanguage pattern mining to induce knowledge models: application in medical image reports knowledge representation. *BMC Med Inform Decis Mak* 2018;**18**:61. doi:10.1186/s12911-018-0645-3

104  Shi L, Ling T, Zhang J. Semantic information extracting system for classification of radiological reports in radiology information system (RIS). In: *Medical Imaging 2016: PACS and Imaging Informatics: Next Generation and Innovations*. San Diego, California, United States: : International Society for Optics and Photonics 2016. 162–75. doi:10.1117/12.2216183

105  Scuba W, Tharp M, Mowery D, *et al.* Knowledge Author: facilitating user-driven, domain content development to support clinical information extraction. *J Biomed Semant* 2016;**7**:42. doi:10.1186/s13326-016-0086-9

106  Monteiro E, Sernadela P, Matos S, *et al.* Semantic Knowledge Base Construction from Radiology Reports. In: *Proceedings of the 9th International Joint Conference on Biomedical Engineering Systems and Technologies HEALTHINF, (BIOSTEC 2016)*. Rome,Italy: : SciTePress 2016. 345–52.https://www.scitepress.org/Link.aspx?doi=10.5220/0005709503450352

107  Hostetter J, Wang K, Siegel E, *et al.* Using Standardized Lexicons for Report Template Validation with LexMap, a Web-based Application. *J Digit Imaging* 2015;**28**:309–14. doi:10.1007/s10278-014-9760-6

108  Short RG, Befera NT, Hoang JK, *et al.* A Normal Thyroid by Any Other Name: Linguistic Analysis of Statements Describing a Normal Thyroid Gland from Noncontrast Chest CT Reports. *J Am Coll Radiol* 2018;**15**:1642–7. doi:10.1016/j.jacr.2018.04.016

109  Xie Z, Yang Y, Wang M, *et al.* Introducing Information Extraction to Radiology Information Systems to Improve the Efficiency on Reading Reports. *Methods Inf Med* 2019;**58**:94–106. doi:10.1055/s-0039-1694992

110  Donnelly LF, Grzeszczuk R, Guimaraes CV, *et al.* Using a Natural Language Processing and Machine Learning Algorithm Program to Analyze Inter-Radiologist Report Style Variation and Compare Variation Between Radiologists When Using Highly Structured Versus More Free Text Reporting. *Curr Probl Diagn Radiol* 2019;**48**:524–30. doi:10.1067/j.cpradiol.2018.09.005

111  Percha B, Zhang Y, Bozkurt S, *et al.* Expanding a radiology lexicon using contextual patterns in radiology reports. *J Am Med Inform Assoc* 2018;**25**:679–85. doi:10.1093/jamia/ocx152

112  Pershad Y, Govindan S, Hara AK, *et al.* Using Naïve Bayesian Analysis to Determine Imaging Characteristics of KRAS Mutations in Metastatic Colon Cancer. *Diagnostics* 2017;**7**:50. doi:10.3390/diagnostics7030050

113  Barbosa F, Traina AJ, Muglia VF. Meta-generalis: A novel method for structuring information from radiology reports. *Appl Clin Inform* 2016;**07**:803–16. doi:10.4338/ACI-2016-03-RA-0037

114  Bulu H, Sippo DA, Lee JM, *et al.* Proposing New RadLex Terms by Analyzing Free-Text Mammography Reports. *J Digit Imaging* 2018;**31**:596–603. doi:10.1007/s10278-018-0064-0

115  Spandorfer A, Branch C, Sharma P, *et al.* Deep learning to convert unstructured CT pulmonary angiography reports into structured reports. *Eur Radiol Exp* 2019;**3**:37. doi:10.1186/s41747-019-0118-1

116  Huesch MD, Cherian R, Labib S, *et al.* Evaluating Report Text Variation and Informativeness: Natural Language Processing of CT Chest Imaging for Pulmonary Embolism. *J Am Coll Radiol* 2018;**15**:554–62. doi:10.1016/j.jacr.2017.12.017

117  Banerjee I, Chen MC, Lungren MP, *et al.* Radiology report annotation using intelligent word embeddings: Applied to multi-institutional chest CT cohort. *J Biomed Inform* 2018;**77**:11–20. doi:10.1016/j.jbi.2017.11.012

118  Shelmerdine SC, Singh M, Norman W, *et al.* Automated data extraction and report analysis in computer-aided radiology audit: practice implications from post-mortem paediatric imaging. *Clin Radiol* 2019;**74**:733.e11-733.e18. doi:10.1016/j.crad.2019.04.021

119  Mabotuwana T, Hombal V, Dalal S, *et al.* Determining Adherence to Follow-up Imaging Recommendations. *J Am Coll Radiol* 2018;**15**:422–8. doi:10.1016/j.jacr.2017.11.022

120  Dalal S, Hombal V, Weng W-H, *et al.* Determining Follow-Up Imaging Study Using Radiology Reports. *J Digit Imaging* 2020;**33**:121–30. doi:10.1007/s10278-019-00260-w

121  Bobbin MD, Ip IK, Sahni VA, *et al.* Focal Cystic Pancreatic Lesion Follow-up Recommendations After Publication of ACR White Paper on Managing Incidental Findings. *J Am Coll Radiol* 2017;**14**:757–64. doi:10.1016/j.jacr.2017.01.044

122  Kwan JL, Yermak D, Markell L, *et al.* Follow Up of Incidental High-Risk Pulmonary Nodules on Computed Tomography Pulmonary Angiography at Care Transitions. *J Hosp Med* 2019;**14**:349–52. doi:10.12788/jhm.3128

123  Mabotuwana T, Hall CS, Tieder J, *et al.* Improving Quality of Follow-Up Imaging Recommendations in Radiology. *AMIA Annu Symp Proc* 2018;**2017**:1196–204.

124  Brown AD, Marotta TR. A Natural Language Processing-based Model to Automate MRI Brain Protocol Selection and Prioritization. *Acad Radiol* 2017;**24**:160–6. doi:10.1016/j.acra.2016.09.013

125  Trivedi H, Mesterhazy J, Laguna B, *et al.* Automatic Determination of the Need for Intravenous Contrast in Musculoskeletal MRI Examinations Using IBM Watson’s Natural Language Processing Algorithm. *J Digit Imaging* 2018;**31**:245–51. doi:10.1007/s10278-017-0021-3

126  Zhang AY, Lam SSW, Liu N, *et al.* Development of a Radiology Decision Support System for the Classification of MRI Brain Scans. In: *2018 IEEE/ACM 5th International Conference on Big Data Computing Applications and Technologies (BDCAT)*. 2018. 107–15. doi:10.1109/BDCAT.2018.00021

127  Brown AD, Marotta TR. Using machine learning for sequence-level automated MRI protocol selection in neuroradiology. *J Am Med Inform Assoc* 2018;**25**:568–71. doi:10.1093/jamia/ocx125

128  Yan Z, Ip IK, Raja AS, *et al.* Yield of CT Pulmonary Angiography in the Emergency Department When Providers Override Evidence-based Clinical Decision Support. *Radiology* 2016;**282**:717–25. doi:10.1148/radiol.2016151985

129  Kang SK, Garry K, Chung R, *et al.* Natural Language Processing for Identification of Incidental Pulmonary Nodules in Radiology Reports. *J Am Coll Radiol* 2019;**16**:1587–94. doi:10.1016/j.jacr.2019.04.026

130  Brown AD, Kachura JR. Natural Language Processing of Radiology Reports in Patients With Hepatocellular Carcinoma to Predict Radiology Resource Utilization. *J Am Coll Radiol* 2019;**16**:840–4. doi:10.1016/j.jacr.2018.12.004

131  Grundmeier RW, Masino AJ, Casper TC, *et al.* Identification of Long Bone Fractures in Radiology Reports Using Natural Language Processing to Support Healthcare Quality Improvement. *Appl Clin Inform* 2016;**7**:1051–68. doi:10.4338/ACI-2016-08-RA-0129

132  Heilbrun ME, Chapman BE, Narasimhan E, *et al.* Feasibility of Natural Language Processing–Assisted Auditing of Critical Findings in Chest Radiology. *J Am Coll Radiol* 2019;**16**:1299–304. doi:10.1016/j.jacr.2019.05.038

133  Maros ME, Wenz R, Förster A, *et al.* Objective Comparison Using Guideline-based Query of Conventional Radiological Reports and Structured Reports. *In Vivo* 2018;**32**:843–9. doi:10.21873/invivo.11318

134  Minn MJ, Zandieh AR, Filice RW. Improving Radiology Report Quality by Rapidly Notifying Radiologist of Report Errors. *J Digit Imaging* 2015;**28**:492–8. doi:10.1007/s10278-015-9781-9

135  Hassanpour S, Langlotz CP. Predicting High Imaging Utilization Based on Initial Radiology Reports:: A Feasibility Study of Machine Learning. *Acad Radiol* 2016;**23**:84–9. doi:10.1016/j.acra.2015.09.014

136  Hassanpour S, Langlotz CP, Amrhein TJ, *et al.* Performance of a Machine Learning Classifier of Knee MRI Reports in Two Large Academic Radiology Practices: A Tool to Estimate Diagnostic Yield. *Am J Roentgenol* 2017;**208**:750–3. doi:10.2214/AJR.16.16128

137  Hsu W, Han SX, Arnold CW, *et al.* A data-driven approach for quality assessment of radiologic interpretations. *J Am Med Inform Assoc* 2016;**23**:e152–6. doi:10.1093/jamia/ocv161

138  Koopman B, Zuccon G, Wagholikar A, *et al.* Automated Reconciliation of Radiology Reports and Discharge Summaries. *AMIA Annu Symp Proc* 2015;**2015**:775–84.

139  Goldshtein I, Chodick G, Kochba I, *et al.* Identification and Characterization of Nonalcoholic Fatty Liver Disease. *Clin Gastroenterol Hepatol* 2020;**18**:1887–9. doi:10.1016/j.cgh.2019.08.007

140  Redman JS, Natarajan Y, Hou JK, *et al.* Accurate Identification of Fatty Liver Disease in Data Warehouse Utilizing Natural Language Processing. *Dig Dis Sci* 2017;**62**:2713–8. doi:10.1007/s10620-017-4721-9

141  Sada Y, Hou J, Richardson P, *et al.* Validation of Case Finding Algorithms for Hepatocellular Cancer from Administrative Data and Electronic Health Records using Natural Language Processing. *Med Care* 2016;**54**:e9–14. doi:10.1097/MLR.0b013e3182a30373

142  Li AY, Elliot N. Natural language processing to identify ureteric stones in radiology reports. *J Med Imaging Radiat Oncol* 2019;**63**:307–10. doi:10.1111/1754-9485.12861

143  Tan WK, Heagerty PJ. Surrogate-guided sampling designs for classification of rare outcomes from electronic medical records data. *ArXiv[Stat.ME]* 2019. http://arxiv.org/abs/1904.00412 (accessed 30 Oct 2020).

144  Yadav K, Sarioglu E, Choi H-A, *et al.* Automated Outcome Classification of Computed Tomography Imaging Reports for Pediatric Traumatic Brain Injury. *Acad Emerg Med* 2016;**23**:171–8. doi:10.1111/acem.12859

145  Mahan M, Rafter D, Casey H, *et al.* tbiExtractor: A framework for extracting traumatic brain injury common data elements from radiology reports. *BioRxiv 585331* 2019. doi:10.1101/585331

146  Brizzi K, Zupanc SN, Udelsman BV, *et al.* Natural Language Processing to Assess Palliative Care and End-of-Life Process Measures in Patients With Breast Cancer With Leptomeningeal Disease. *Am J Hosp Palliat Med* 2019;**37**:371–6. doi:https://doi.org/10.1177/1049909119885585

147  Van Haren RM, Correa AM, Sepesi B, *et al.* Ground Glass Lesions on Chest Imaging: Evaluation of Reported Incidence in Cancer Patients Using Natural Language Processing. *Ann Thorac Surg* 2019;**107**:936–40. doi:10.1016/j.athoracsur.2018.09.016

148  Noorbakhsh-Sabet N, Tsivgoulis G, Shahjouei S, *et al.* Racial Difference in Cerebral Microbleed Burden Among a Patient Population in the Mid-South United States. *J Stroke Cerebrovasc Dis* 2018;**27**:2657–61. doi:10.1016/j.jstrokecerebrovasdis.2018.05.031

149  Gould MK, Tang T, Liu I-LA, *et al.* Recent Trends in the Identification of Incidental Pulmonary Nodules. *Am J Respir Crit Care Med* 2015;**192**:1208–14. doi:10.1164/rccm.201505-0990OC

150  Huhdanpaa HT, Tan WK, Rundell SD, *et al.* Using Natural Language Processing of Free-Text Radiology Reports to Identify Type 1 Modic Endplate Changes. *J Digit Imaging* 2018;**31**:84–90. doi:10.1007/s10278-017-0013-3

151  Bates J, Fodeh SJ, Brandt CA, *et al.* Classification of radiology reports for falls in an HIV study cohort. *J Am Med Inform Assoc* 2016;**23**:e113–7. doi:10.1093/jamia/ocv155

152  Masino AJ, Grundmeier RW, Pennington JW, *et al.* Temporal bone radiology report classification using open source machine learning and natural langue processing libraries. *BMC Med Inform Decis Mak* 2016;**16**:65. doi:10.1186/s12911-016-0306-3

153  Valtchinov VI, Lacson R, Wang A, *et al.* Comparing Artificial Intelligence Approaches to Retrieve Clinical Reports Documenting Implantable Devices Posing MRI Safety Risks. *J Am Coll Radiol* 2020;**17**:272–9. doi:10.1016/j.jacr.2019.07.018

154  Charles E. Khan Jr. An Ontology-Based Approach to Estimate the Frequency of Rare Diseases in Narrative-Text Radiology Reports. In: *MEDINFO:2017 Precision Healthcare through Informatics*. IOS Press Ebooks:896–900.http://ebooks.iospress.nl/publication/48282

155  Zech J, Forde J, Titano JJ, *et al.* Detecting insertion, substitution, and deletion errors in radiology reports using neural sequence-to-sequence models. *Ann Transl Med* 2019;**7**. doi:10.21037/atm.2018.08.11

156  Zhang Y, Merck D, Tsai EB, *et al.* Optimizing the Factual Correctness of a Summary: A Study of Summarizing Radiology Reports. *ArXiv[Cs.CL]* 2019. http://arxiv.org/abs/1911.02541 (accessed 30 Oct 2020).

157  Steinkamp JM, Chambers C, Lalevic D, *et al.* Toward Complete Structured Information Extraction from Radiology Reports Using Machine Learning. *J Digit Imaging* 2019;**32**:554–64. doi:10.1007/s10278-019-00234-y

158  Cocos A, Qian T, Callison-Burch C, *et al.* Crowd control: Effectively utilizing unscreened crowd workers for biomedical data annotation. *J Biomed Inform* 2017;**69**:86–92. doi:10.1016/j.jbi.2017.04.003

159  Koza W, Filippo D, Cotik V, *et al.* Automatic Detection of Negated Findings in Radiological Reports for Spanish Language: Methodology Based on Lexicon-Grammatical Information Processing. *J Digit Imaging* 2019;**32**:19–29. doi:10.1007/s10278-018-0113-8

160  Peng Y, Wang X, Lu L, *et al.* NegBio: a high-performance tool for negation and uncertainty detection in radiology reports. *AMIA Summits Transl Sci Proc 2018* 2018;**2017**:188–96.

161  Sergeeva E, Zhu H, Prinsen P, *et al.* Negation Scope Detection in Clinical Notes and Scientific Abstracts: A Feature-enriched LSTM-based Approach. *AMIA Summits Transl Sci Proc* 2019;**2019**:212–21.

162  Cotik V, Stricker V, Vivaldi J, *et al.* Syntactic methods for negation detection in radiology reports in Spanish. Berlin, Germany: : Association for Computational Linguistics 2016. 156–65. doi:10.18653/v1/W16-2921

163  Chen H, Gangaram V, Shih G. Developing a More Responsive Radiology Resident Dashboard. *J Digit Imaging* 2019;**32**:81–90. doi:10.1007/s10278-018-0123-6

164  Yetisgen M, Klassen P, McCarthy L, *et al.* Annotation of Clinically Important Follow-up Recommendations in Radiology Reports. In: *Proceedings of the Sixth International Workshop on Health Text Mining and Information Analysis*. Lisbon, Portugal: : Association for Computational Linguistics 2015. 50–54. doi:10.18653/v1/W15-2606
